# Multitasking While Driving: Central Bottleneck or Problem State Interference?

**DOI:** 10.1177/00187208221143857

**Published:** 2022-12-06

**Authors:** Moritz Held, Jochem W. Rieger, Jelmer P. Borst

**Affiliations:** Bernoulli Institute for Mathematics, Computer Science and Artificial Intelligence, 3647 University of Groningen, The Netherlands; Department of Psychology, University of Oldenburg, Germany; Department of Psychology, University of Oldenburg, Germany; Bernoulli Institute for Mathematics, Computer Science and Artificial Intelligence, 3647 University of Groningen, The Netherlands

**Keywords:** cognitive modeling, adaptive automation, human computer interaction, mental workload, driver behavior

## Abstract

**Objective:**

The objective of this work was to investigate if visuospatial attention and working memory load interact at a central control resource or at a task-specific, information processing resource during driving.

**Background:**

In previous multitasking driving experiments, interactions between different cognitive concepts (e.g., attention and working memory) have been found. These interactions have been attributed to a central bottleneck or to the so-called problem-state bottleneck, related to working memory usage.

**Method:**

We developed two different cognitive models in the cognitive architecture ACT-R, which implement the central vs. problem-state bottleneck. The models performed a driving task, during which we varied visuospatial attention and working memory load. We evaluated the model by conducting an experiment with human participants and compared the behavioral data to the model’s behavior.

**Results:**

The problem-state-bottleneck model could account for decreased driving performance due to working memory load as well as increased visuospatial attentional demands as compared to the central-bottleneck model, which could not account for effects of increased working memory load.

**Conclusion:**

The interaction between working memory and visuospatial attention in our dual tasking experiment can be best characterized by a bottleneck in the working memory. The model results suggest that as working memory load becomes higher, drivers manage to perform fewer control actions, which leads to decreasing driving performance.

**Application:**

Predictions about the effect of different mental loads can be used to quantify the contribution of each subtask allowing for precise assessments of the current overall mental load, which automated driving systems may adapt to.

## INTRODUCTION

Driving is one of the most complex tasks that people do on a daily basis. In order to safely navigate traffic, drivers must monitor in-vehicle controls such as the speedometer or navigation devices while also paying attention to the surroundings and watching out for other road users and pedestrians. Thus, it is unsurprising that an estimated 94% of motor vehicle crashes can be attributed to human error (Highway Traffic Safety Administration & Department of Transportation, 2016). One of the main sources of human error while driving is cognitive overload (e.g., Engström et al., 2017), next to distraction (e.g., Hancock et al., 2003) and fatigue (e.g., Gunzelmann et al., 2011). However, the effect of cognitive workload on driving is not straightforward and depends on many factors such as the specific kind and amount of workload as well as driving difficulty.

### Cognitive Workload While Driving

Researchers investigating cognitive workload during driving often focus on visual distractors that impose additional workload on the drivers as it is considered central to driving. Consequently, the literature of the negative effects of extended visual distraction on driving performance is comprehensive (e.g., Hancock et al., 2003; Horberry et al., 2006; Ito et al., 2001; Li et al., 2018; Papantoniou et al., 2017). However, even when secondary tasks impose only minimal visual demands, driving performance has been shown to suffer as well (Alm & Nilsson, 1995; Jenness et al., 2002; Tsimhoni et al., 2004) suggesting that driving draws on multiple cognitive resources, which are affected by cognitive distractions.

Similar to visual distractors drawing the eyes away from the road, which exploit the limitations of human sensing, cognitive distractors take away cognitive resources from the driving task and interfere with the driving task at the level of information processing. For example, Strayer et al. (2015) induced high cognitive workload by a variety of different factors such as engaging in a conversation or operating a speech-to-text system to reply to text messages. They found that higher mental demand of the secondary task, as measured by NASA-TLX ratings (Hart & Staveland, 1988), can be linked to longer brake reaction times in a car-following task as well as to worse scanning behavior for hazard locations. Another study found lower situational awareness and a higher number of driving infractions (e.g., missing stop sign) while engaging in a phone conversation while driving (Kass et al., 2007).

Contrary to intuition, slightly increased levels of cognitive workload can also improve driving performance when the driving task is monotonous (Nijboer et al., 2016). Nijboer and colleagues (2016) attempted to reconcile these results by proposing that cognitive workload may have a preventative effect when mundane driving situations might otherwise lead to mind-wandering or fatigue, which have been associated with poor driving performance (e.g., Gunzelmann et al., 2011; Martens & Brouwer, 2013). Results showing that this effect disappears when driving difficulty is varied provide additional support for this explanation (Engström et al., 2017; Medeiros-Ward et al., 2014). While the beneficial effect of additional load in monotonous driving situations can be found using a variety of different secondary tasks such as interactive verbal tasks (Atchley & Chan, 2011), n-back tasks (Medeiros-Ward et al., 2014), or simply repeating a sequence of numbers in reverse order (He & McCarley, 2011) in lab studies, it is not as consistent in naturalistic driving situations (Engström et al., 2017).

The complexity of the interaction is further highlighted by studies of Unni and colleagues (2017) and Scheunemann and colleagues (2019) who attempted to classify the cognitive workload of drivers from fNIRS data in a realistic driving simulator. The authors manipulated both visuospatial demands through different lane width and working memory load induced by a modified n-back task. Guided by Wickens’ model of multiple resources (Wickens, 2002), the authors predicted that both tasks required a different set of cognitive resources and expected little interference of one task on the other. Following that line of reasoning, Unni and colleagues (2017) attempted to classify the working memory load independent of visuospatial demands from the recorded fNIRS data using multivariate regression. They found high correlations between the predicted workload and the induced workload. However, when attempting to reversely classify visuospatial demands independent of working memory load levels, Scheunemann and colleagues (2019) only reached low classification accuracies. In a follow-up analysis, the authors showed high classification accuracy when the classification was done for individual working memory load levels (e.g., intermediate working memory load) demonstrating brain-level interactions between the two manipulated cognitive concepts. Theories of multitasking can account for this finding in different ways. The authors posited as one option that the two tasks might not be separated to different cognitive resources and share a common resource specific to the two tasks, which according to Wickens’ model of multiple resources (Wickens, 2002) would incur a significant cost of multitasking. Alternatively, the two tasks might interact at a task-unspecific level instead. Both Wickens’ model (at the central executive) as well as the often observed effect of the psychological refractory period (PRP) (Pashler, 1994; Welford, 1952) similarly predict a performance loss when multitasking due to a bottleneck at a central resource (Levy et al., 2006).

The goal of this study was to identify the mechanisms underlying the observed interaction between visuospatial demands and working memory load (Scheunemann et al., 2019). While decoding models such as the one used by Scheunemann and colleagues (2019) are useful to indicate where task-relevant information processing is located in the brain, these models are limited when trying to explain the functional mechanisms that drive task performance (Kriegeskorte & Douglas, 2018). As an alternative, cognitive models are particularly useful in this regard as they permit insight into *how* people are performing a task. Cognitive modeling is a widely established method to understand human cognition and, moreover, to create explainable computational models, which can be used not only to evaluate hypotheses about task strategies but also to determine how each task depends on available cognitive resources (Kriegeskorte & Douglas, 2018; Newell, 1973).

To elucidate the effects of workload on driving, we adapted the seminal driving model by Salvucci (2006) designed in the Adaptive Control of Thought—Rational (ACT-R; Anderson, 2007) cognitive architecture. We contrasted two different bottleneck models, which have both been related to considerable interference in multitasking situations: (1) A central-bottleneck model, where interference is due to competition for a central coordination system (Pashler, 1994; Salvucci & Taatgen, 2008; Taatgen et al., 2009) and (2) a problem-state-bottleneck model, where interference is due to contention of a working memory resource (Borst et al., 2010; Nijboer et al., 2016). To test which of these models can better account for cognitive interactions in driving, we conducted an experiment in which two cognitive concepts often utilized in driving situations were manipulated, working memory, and visuospatial attention (Scheunemann et al., 2019; Unni et al., 2017).

### Modeling Approach

To investigate the interaction described by Scheunemann et al. (2019), we developed two cognitive models in the cognitive architecture ACT-R. ACT-R is a psychological theory of human cognition implemented as a computer simulation (Anderson, 2007). It aims to incorporate the basic cognitive processes that enable the human mind and can model cognitive processes not as a single operation but as part of a coherent system that produces all of human behavior. It allows for the development of cognitive models, which can be understood as an implementation of a hypothesis of how humans solve a particular task. ACT-R has been used to develop one of the most well-known driving models (Salvucci, 2006) and, furthermore, has been used in previous studies to model visual sampling on in-car displays (Kujala & Salvucci, 2015), and to simulate the effects of memory rehearsal (Salvucci & Beltowska, 2008) and phone dialing (Salvucci & Macuga, 2002) on driving performance.

**Figure 1.**
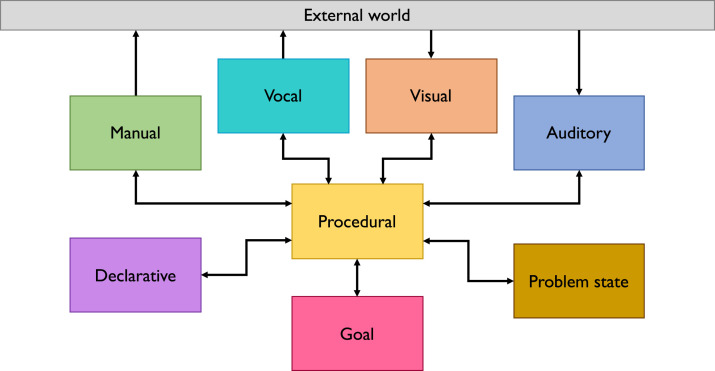
ACT-R cognitive architecture.

ACT-R simulates human cognition by a set of modules and buffers, which are each specialized to process a specific kind of information. How available information is processed, is determined by how the information is passed through the modules of ACT-R, which communicate with each other via their respective buffers and are coordinated by a central production system (Figure 1). A production is a specific action formulated as an if-then rule that takes 50 ms to execute. For example, *if* the visual module perceives a speed sign, *then* the speed can be either stored in the problem state or in declarative memory. Thus, cognitive processes are expressed as a series of productions, which each occupy a module for a certain amount of time. Productions are executed by the procedural module, which makes up the procedural knowledge of the model.

Next to the procedural module, other relevant modules for driving are the declarative memory module, the goal module and the problem state. Declarative memory makes up long-term knowledge of ACT-R and contains chunks of information with slot-value pairs. Chunks can be manually defined or learned by the model during the experiment. Each chunk has a certain activation level that is based on the recency (i.e., when the chunk has last been processed) and frequency (i.e., how often the chunk been processed). The activation *B*_*i*_ of a chunk *i* continuously decays following the equation

$$B_{i}=\ln\left(\overset{n}{\underset{j=1}{\sum }}t_{j}^{-d}\right),$$

where *n* denotes the total number of presentations of chunk *i*, *t*_*j*_ denotes the time since the *jth* presentation of said chunk, and finally, *d* represents a decay parameter usually set to 0.5. To successfully retrieve a chunk, the activation has to be above the retrieval threshold, which is set by the modeler. By clearing the buffer of any module, the chunk currently in that buffer is stored in declarative memory—thereby essentially creating new memories.

The problem state holds the current information that is necessary to carry out the task; it serves as a one-chunk working memory, reflecting the focus of attention in modern working memory theories (Borst et al., 2010; Nijboer et al., 2016). The goal module contains the control information needed to perform the task. Thus in a real task, the goal module would, for example, hold the goal information that upcoming speed signs need to be attended, the visual module would perceive and attend speed signs, and the encoded speeds would be stored in the problem state. Upon entering the problem state, a retrieval to a previous sign could be issued, for example, to check if the new speed sign is the same as the previous. If there is a chunk in declarative memory above the retrieval threshold which matches the description of the retrieval attempt, the chunk would enter the declarative memory buffer upon which it can be accessed by other modules.

ACT-R is limited by the central processing unit, which can only initiate one production at a time. When the conditions for two productions are met at the same time, only one of them can be executed at once, resulting in a processing bottleneck. At the same time, multiple processes may run in parallel when separated to different modules—for example, a memory retrieval can be made while the model focuses on a speed sign—but each module can only deal with one instruction at a time (Byrne & Anderson, 2001). This limited parallelism has strong implications for multitasking, as it not only leads to a high contention for the central processing unit, but also implies that tasks that are reliant on different modules can be performed more efficiently than others.

On top of the inherent multitasking behavior of ACT-R, Salvucci and Taatgen (2008, 2011) developed the multitasking theory *threaded cognition*, which is integrated into ACT-R. Threaded cognition specifies further how available resources are used in pursuit of multiple task goals. It allows the model to interleave two or more tasks by using a greedy and polite principle which dictates that productions occupy modules as quickly as possible but also release the modules as soon as they are not needed. Production rules will simply be executed based on the availability of modules and buffers independently of their urgency. If two production rules of different task goals can be executed, the production of the least recently attended goal will be selected. Since its inception, threaded cognition has been used successfully in a variety of multitasking settings and has been established as a sophisticated multitasking theory that can account for a range of multitasking behavior (e.g., Borst et al., 2010; Kujala & Salvucci, 2015; Salvucci & Beltowska, 2008) and associated brain activity (e.g., Borst et al., 2011; Borst et al., 2010; Nijboer et al., 2014). Due to its management of multiple resources, threaded cognition is often regarded as a computational implementation of Wickens’ model of multiple resources (Wickens, 2002).

Inspired by the work of Salvucci and Beltowska (2008) who implemented a memory task while driving and matched the model to human behavior, we developed two ACT-R models. In the first model, we relied on the inherent multitasking behavior of ACT-R, which is limited by the serial initiation of production rules and thereby modeled a bottleneck at a task-unspecific resource (central bottleneck), which is independent of the specific tasks of the model. In the second model, the multitasking behavior was characterized by a dependency on the problem state during multitasking and thereby implemented a bottleneck at a task-specific resource (problem-state bottleneck). By comparing the behavior of the two models, we contrasted the two possible hypotheses posited by Scheunemann et al. (2019). Both models performed an experiment designed by Unni et al. (2017). To allow for better validation of the model, we collected new human data with this experimental setup. Furthermore, we used eye-tracking to assess cognitive workload of the human participants.

## METHOD

### Participants

25 participants (12 male, 13 female) aged between 20 and 37 years (mean = 26; SD = 4.1) were recruited in the Oldenburg area, which was a similar sample size as in previous studies (e.g., Unni et al., 2017). All participants were in possession of a valid driver’s license in Germany at the time of the experiment. They gave informed consent prior to the experiment and received 10€/hour as a reimbursement. This research complied with the American Psychological Association Code of Ethics and this experiment was approved by the Ethics Committee of the Carl von Ossietzky University Oldenburg. The data of three participants had to be excluded due to a technical error with the eye-tracker. Average age of the included participants was 26.2 (SD = 4.3).

## Materials

The driving experiment was programmed in Java using the code framework of previous research by Kujala and Salvucci (2015) and made public at: https://www.cs.drexel.edu/~salvucci/cog/act-r/download.php. It was conducted in the behavioral lab of the Applied Neurocognitive Psychology Laboratory at the University of Oldenburg. It was run on a 1920x1080 px monitor in a Microsoft Windows 10 environment in combination with a Logitech G20 wheel, which included a throttle and brake pedal. Experimental data (e.g., car position) were sampled with a rate of 200 Hz.

A Portable EyeLink Duo 2 (SR Research Ltd., Ottawa, Canada) eye-tracker was used to track gaze position and pupil dilation with a sampling rate of 500 Hz. For this set-up, we selected nine-point calibration with mono-ocular tracking (left eye) in remote tracking mode.

### Design

**Figure 2.**
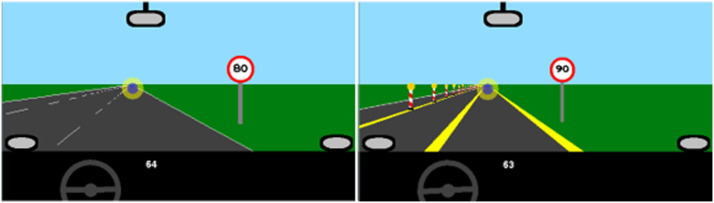
Road conditions in the highway condition (left) and the construction condition (right). The circle indicates the visual focus of the model (not visible to participants).

The experiment used a within-subject design which varied the factors visuospatial attention and working memory load. Adapted from Unni and colleagues (2017) and Scheunemann and colleagues (2019), visuospatial attention requirements were manipulated by either driving on a highway with three lanes of 3.5 m each or through a construction site with lanes of 2.5 m each. In the construction site, the left-most lane was blocked off by red-white pylons (Figure 2). The participants were instructed to stay in the middle of their current lane and drive on the right-most lane that was not occupied by another vehicle. In both conditions, the road was completely straight with no bends to either side.

**Figure 3.**
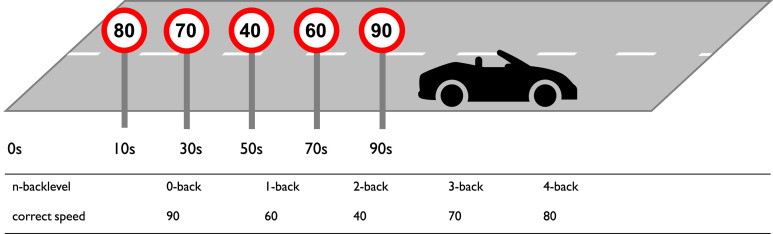
Example of an n-back block at the 90s mark. Bottom row displays the appropriate speed at the car’s position depending on the n-back level.

Working memory load was manipulated by a modified n-back task. Participants were instructed to watch out for speed signs which appeared on the right side of the road. The first speed sign appeared 10 seconds in to the block, subsequent signs appeared every 20 seconds thereafter. The signs displayed a speed limit of base 10 between 40 km/h and 120 km/h. Participants were instructed to drive according to the speed limit that was displayed *n* signs ago. To keep to the task-compliant speed, participants had to monitor the surrounding traffic on adjacent lanes and the available mirrors in order to perform overtaking maneuvers which became necessary ca. 2–3 times per block. Five different n-back levels were used, ranging from 0-back during which participants simply followed the latest speed sign (i.e. regular driving) to 4-back during which participants had to keep their speed in accordance with the speed sign that they saw four signs ago (Figure 3). To be able to successfully perform this task, *n* speed signs needed to be passed before the experimental phase could start. This build-up phase of the complete to-be-memorized sequence preceded the experimental phase of each block and was not analyzed.

### Procedure

Upon arrival, participants were seated and the eye-tracking equipment was calibrated. Afterward, participants started with a 5-minute training session, during which they performed a 2-back task in the highway condition to get used to the driving environment and vehicle controls. If participants were not comfortable with the controls after the training session or did not understand the instructions of the n-back task, we repeated the training session. Before starting the experiment, we applied a drift correction of the eye-tracker and re-calibrated if necessary.

The main experiment lasted ca. 70 minutes with a break in the middle. Each block of the experiment lasted exactly 160 s of experimental phase in addition to the build-up phase (see above). For the first half of the experiment the n-back levels were each paired once with either visuospatial condition such that visuospatial condition altered between every block and no n-back level would occur twice in a row. This set of ten blocks was then repeated in reverse order for the second half of the experiment resulting in a total number of 20 blocks (following Scheunemann et al., 2019; Unni et al., 2017).

### Analysis

The build-up phase was excluded for all analyses from both human and model data. One block for one participant was additionally excluded due to disregarding the instructions, which was indicated by the participant remaining stationary during the first minutes of the block. Driving segments during which a lane-change maneuver occurred were discarded. These segments were defined by a 6 second interval around a crossing of the lane markings (Nijboer et al., 2016). To avoid classifying lane keeping errors as lane-changes, only segments during which participants drove for at least 5 seconds in the adjacent lane after crossing the lane marking were considered as lane-changes.

As a measurement for driving performance, we calculated steering reversal rates which have been indicated to increase with increased effort in the driving task (Greenshields, 1963; Hicks & Wierwille, 1979; Macdonald & Hoffmann, 1977; McLean & Hoffmann, 1975; Scheunemann et al., 2019). Steering reversals were defined as a crossing of the steering wheel’s center position (i.e., going from left to right or vice versa). Errors in the speed regulation task were difficult and inefficient to evaluate automatically as participants showed vastly different acceleration patterns when encountering a speed sign. Thus, the trials were each manually inspected and rated as incorrect when participants failed to reach and maintain the correct speed with a tolerance of ± 5 km/h on average. In addition to steering reversals, we also calculated the average lane deviation. Lane deviation was defined as the mean of the absolute distance to the center of the currently occupied lane.

Before analyzing pupil dilation as an indicator for cognitive workload (Kahneman & Beatty, 1966), the data were pre-processed by removing eye-blinks, which were identified by the integrated blink detection provided by SR Research. Other artifacts (e.g., due to temporary eye-tracker failures) were identified as being rapid changes in pupil sizes to values at least four standard deviations away from the mean. They were corrected by removing all samples from 25 samples (50 ms) before until 25 samples after the blink and replacing the samples by monotonic cubic spline interpolation following the recommendations of Mathôt and colleagues (2018). Afterward, we calculated the percentage change to a baseline period, which was determined to be the time window of 5 seconds after the start of the experiment and 5 seconds before the occurrence of the first speed sign. The baseline was calculated as the median pupil size of the fixations during the baseline period (Mathôt et al., 2018). Furthermore, we calculated the average percentage change for pupil size of the fixations per unique combination of n-back level and visuospatial condition. Fixations were determined via the integrated software by SR Research, which categorized a fixation as a temporary spatially stable gaze direction.

**Figure 4.**
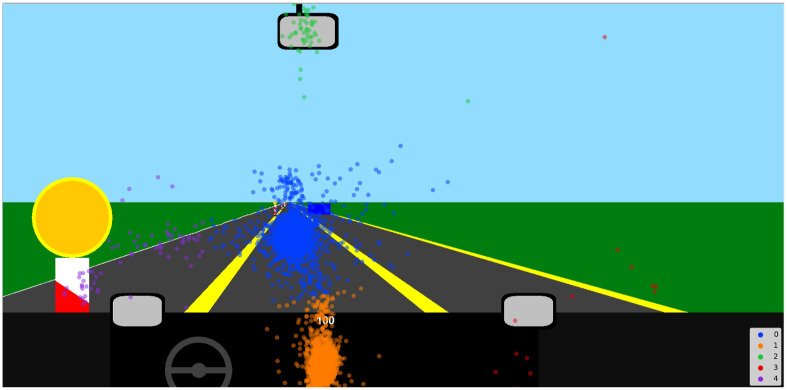
Clustering of fixations shown on example participant (Participant 5). Clusters 0, 1, 2, 4 can be assigned to specific locations (upcoming road, speedometer, rear-view mirror, and left lane, respectively). Cluster no. 3 cannot be assigned to a location and accounts for noise.

For the analysis of gaze position, we clustered the fixations with k-means clustering. We selected the number of the clusters manually due to the vast differences regarding participants’ use of mirrors and determined the number for each participant and visuospatial condition. The number of clusters was deemed appropriate when at least *k*−1 clusters could be assigned a distinct location (e.g., three clusters: speedometer, road, mirror) with one optional cluster accounting for noise (see Figure 4 for a demonstration of this method). Labels were assigned manually. Afterward, we calculated the average number of fixations on the speedometer in between two speed signs for each combination of visuospatial and n-back combination.

### Model

The model used in this study was a combination of a driving model and an n-back model. The driving model was a modification of the Salvucci driving model (Salvucci, 2006), which is able to drive on an empty highway. However, as the original model does not account for effects of different lane widths, we adapted the model for this study.

### Driving Model

For lateral control, that is maintaining a smooth trajectory in the center of the lane, we implemented a system with a high-control and a low-control loop. The high-control loop closely resembles the model of (Salvucci, 2006), which employs the two-point steering model described by Salvucci and Gray (2004). The model negotiates a new steering angle at every iteration of the loop based on a near point and far point at the lane center, which are 10 m and 100 m in front of the car, respectively.

The low-control loop follows a different strategy that does not update the steering angle until necessary and thereby models a more passive control mechanism. Here, the model maintains the same steering angle until the car comes close to the edge of a lane. Once that happens, the model switches back to the high-control loop to safely move back to the center.

Both the minimal lateral distance to the lane edge that triggers the high-control loop (*dist*) and the time the model stays in the high-control loop (*t*_*hc*_) to move back to the center of the lane are parameters which affect driving performance. As more time in the high-control loop equates to a tighter control of the steering resulting in more steering corrections, the parameters have been adjusted for a good fit of the model’s steering behavior (see Appendix, Table A1).

**TABLE 1:** Example of a memory chunk

Slot	Value
Isa	nback
ID	80
previousID	60
Speed limit	70

For longitudinal control, the model continuously accelerates and decelerates to maintain a fixed distance to a point moving at the target speed. The control law of the model utilizes to achieve this was based on the control law by, Salvucci (2006)

$$\Delta \Phi =k_{\Delta thw}\cdot \Delta thw+k_{thw}\cdot thw\Delta t,$$

where Φ denotes an acceleration value ranging from −1, which translates to maximally pressing down the brake, to 1, which translates to maximally pressing down the throttle and ΔΦ denotes the difference between two acceleration values between two iterations of the driving loop. At the value of 0, neither throttle nor brake are pressed down. *thw* describes the time headway between the car and a fictional point in the distance, which moved according to the speed limit and Δ*thw* the difference between two iterations of the driving loop. In other words, the distance from the car to the distant point is defined as the distance an object travels in the time interval Δ*t* when following the speed limit. Δ*t* describes the time difference between the last update of the model and the current time *t*.

Additional adaptations of the model include overtaking surrounding cars by changing the near and far point to the adjacent lane (following Salvucci, 2006) while using the side and rear-view mirrors to monitor the cars in the adjacent lanes and driving in the right-most lane whenever possible.

### N-Back Model

The part of the model performing the modified n-back task works via a sequential recall mechanism. As soon as a speed sign appears, it is encoded with an episodic tag as a single chunk and released to the declarative memory of ACT-R (Table 1). Episodic tags (ID in Table 1) are necessary to avoid the merging of speed signs that display the same speed at different moments of the experiment as well as to ensure that the speed signs are encoded in the correct order. Additionally, the chunks contain a slot with the episodic tag of the previous speed sign (previousID in Table 1). Thus, the encoding of the speed signs can be described as a linked list going backwards in time. To successfully recall a sign, the model sequentially goes through the speed signs starting at the most recent speed sign and ending at the *nth* sign depending on the n-back level of the current task. For each step going backwards, the model initiates a retrieval request for a chunk carrying the episodic tag of the previous sign. This episodic tag is stored in a chunk in the problem state buffer. When the chunk of the previous sign is retrieved from memory, it replaces the chunk in the problem state buffer causing the chunk of the current sign to be released to declarative memory. As rehearsal is a prominent strategy to accomplish the n-back task in humans, the sequence of task-relevant speed signs is rehearsed via the same sequential mechanism, after successful recall (Chooi & Logie, 2020).

**TABLE 2:** Similarity of speed signs appearing at different times to the exemplary chunk in Table 1, which appeared at 80 seconds

Time	20	40	60	80	100	120	140	160	…
Similarity	−0.3	−0.2	−0.1	0	−0.1	−0.2	−0.3	−0.4	…

Due to the limitations in multitasking in ACT-R models, which reflect human limitations, the number of rehearsals affected driving performance, because the steering updates compete with the initiation of productions of the rehearsal. As a consequence, we adjusted the number of rehearsals (analogous to Salvucci & Taatgen, 2011, Chapter 4) for a good model fit of the participants behavior (see Table A1 in Appendix).

It seems unlikely that speed signs were completely forgotten. Therefore, the retrieval threshold *rt* was set to a low value. Errors are modeled by partial matching, which can be thought of as mixing up two signs (Lebiere, 1999). How easily a chunk can be erroneously retrieved instead of the matching chunk is determined by the similarity between the two chunks. The similarity was chosen to be linearly decreasing with the time difference when two speed signs appeared. For example, two consecutive speed signs are more similar than two speed signs with a 1 minute time difference in between. Similarity ranged from 0 meaning that two chunks encoded the same speed sign and decreased by −0.1 for each speed sign that occurred in between (Table 2). The experiment and the two driving models below are available under: https://github.com/ANCPLabOldenburg/.

**TABLE 3:** Cost of the two bottlenecks

	Problem-State Bottleneck		Central Bottleneck	
N-back	Construction	Highway	Construction	Highway
0-back	0.006	0.009	0.151	0.160
1-back	0.345	0.272	0.526	0.543
2-back	0.518	0.515	0.664	0.682
3-back	2.205	1.865	0.835	0.841
4-back	4.941	4.406	0.971	0.995

### Multitasking

**Figure 5.**
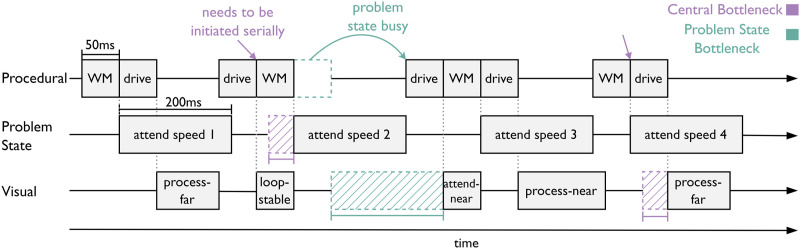
Demonstration of the two different bottlenecks. The two goals, WM (working memory) and drive, are initiated by the procedural module.

Multitasking was modeled by employing two goals (driving and the n-back task), which are continuously interleaved using threaded cognition (Salvucci & Taatgen, 2008). Salvucci and Beltowska (2008) identified a bottleneck at the central processing unit of ACT-R when two tasks are being performed simultaneously, which negatively affected driving performance. In the central-bottleneck model, we did not explicitly model an interaction between the driving part of the model and the n-back part of the model. Instead, following the work of Salvucci and Beltowska (2008), the model is based on the competition for the processing unit of ACT-R. Because only one production rule can be initiated at any one time and its execution takes 50 ms, it can cause delays in either task when multiple production rules have their conditions met at the same time. For example, in Figure 5, purple arrows indicate when productions can be initiated for both the driving task and n-back task as all resources are available. As the production rule of the least recently attended goal gets initiated first, this delays the productions of the other task (purple dashed lines in Figure 5), which in the case of driving can result in fewer steering updates, when n-back productions are initiated first (Anderson et al., 2005).

In the problem-state-bottleneck model, we used the same driving and n-back mechanisms as in the central-bottleneck model. However, we made the multitasking requirement stronger by introducing a dependency on working memory (i.e. the problem state) into the driving task. In concrete terms, this meant that each iteration of the high- or low-control loop, which starts with a so-called “attend-near”-production, could only be initiated when the problem state was not busy (green arrow in Figure 5 indicates the delay). As a consequence, no new steering actions are initiated while working memory is actively used. As the sequential recall of speed signs for the n-back task requires constant swapping of the contents of the problem state buffer, it will remain active for longer periods during the task (i.e., 200 ms after each recall). This mechanism results in significant delays regarding the initiation of the driving loop as it falls more often together with a swap of the content of the problem state (green dashed lines in Figure 5. Note that, as the bottleneck in the central processing unit is an inherent feature of the multitasking behavior of ACT-R, it was also part of the problem-state-bottleneck model. We re-fit the driving parameters (see Table A1 in Appendix) to ensure that delayed control operations due to the restrictions in multitasking did not cause the model to lose control of the vehicle.

## RESULTS

### Driving Performance

**Figure 6.**
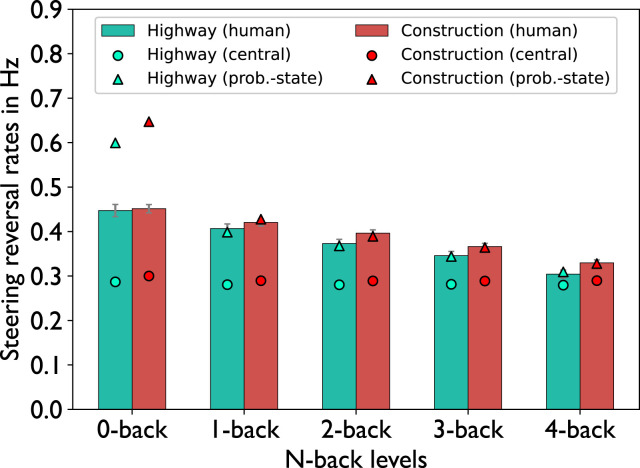
Average steering reversal rates. Error bars indicate the standard errors of the mean.

In line with previous studies, for human drivers we hypothesized an effect of working memory load (n-back level) and driving difficulty (narrow vs. wide lane) on driving behavior measured by steering reversal rate (SRR) and lane deviation (De Waard & Brookhuis, 1996). Figure 6 shows the significant decrease in SRRs in human participants that we observed across all n-back levels $\left(F\left(4,84\right)=38.4,p<0.001,\eta_{p}^{2}=0.65\right)$ as well as a significant increase in SRRs in the construction condition compared to the highway condition $\left(F\left(1,21\right)=16.3,p<0.001,\eta_{p}^{2}=0.44\right)$ , which was supported by a two-factor repeated measures analysis of variance (ANOVA) with the factors n-back level and lane width. There was no interaction effect $\left(F\left(4,84\right)=1.5,p=0.22,\eta_{p}^{2}=0.07\right)$ .

Table 3 shows the “cost” of the two bottlenecks as the average sum of all production delays in between two speed signs due to the respective bottlenecks. As the n-back level increases, the cost increases for both bottlenecks. However, as the central-bottleneck is solely impacted by the increased competition for the procedural memory, it shows a lower increase and a lower total cost when compared to the problem-state bottleneck. Importantly, the problem-state-bottleneck model is impacted by both bottlenecks whereas the central-bottleneck model is merely restricted by the central bottleneck.

**TABLE 4:** Formal model fit metrics

	RMSE	*R* ^2^
SRR (central)	0.11	0.31
SRR (problem state)	0.08	0.77
Lane deviation (central)	0.22	0.92
Lane deviation (problem state)	0.15	0.14
Errors (central	0.08	0.95
Errors (problem state)	0.07	0.83

The problem-state-bottleneck model predicted both significant effects regarding n-back level and lane width, and matched the human data closely. This shows that, with respect to SRR prediction, the problem-state-bottleneck model (triangles in Figure 6) can clearly account for both effects. We calculated the root mean square error (RMSE) (Chai & Draxler, 2014) and the determination coefficient *R*^2^ to formally evaluate the models and calculated an *RMSE* = 0.08 and an *R*^2^ = 0.77 for the problem-state-bottleneck model (see Table 4). However, the central-bottleneck model (circles in Figure 6), while steering slightly more often in the construction condition and thereby reflecting the human behavior, 1) underestimated this effect and—more importantly—2) was not able to capture the effect of decreasing SRRs across n-back levels (*RMSE* = 0.11, *R*^2^ = 0.31).

**Figure 7.**
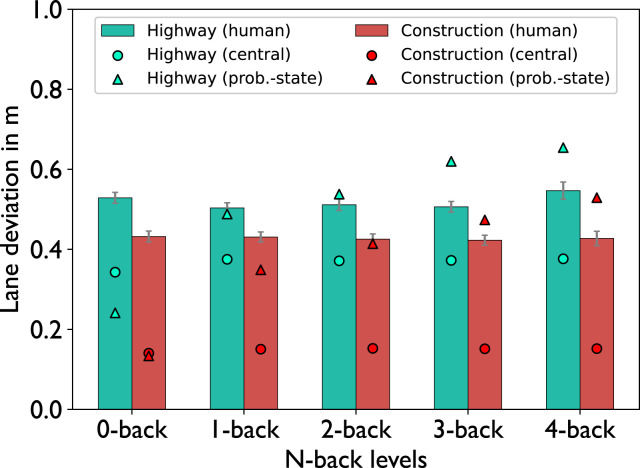
Average lane deviation. Error bars indicate the standard errors of the mean.

Analysis of lane deviation (Figure 7) showed that human participants deviated more from the lane center in the highway condition than in the construction condition $\left(F\left(1,21\right)=81.2,\;p<0.001,\eta_{p}^{2}=0.79\right)$ . Although there was no main effect of n-back difficulty $\left(F\left(4,84\right)=0.19,p=0.94,\eta_{p}^{2}=0.01\right)$ , the two-factor repeated measures ANOVA revealed a significant interaction effect $\left(F\left(1,21\right)=3.3,p=0.0156,\eta_{p}^{2}=0.13\right)$ , where the difference between highway and construction increased with increasing n-back level. This interaction seems to be mostly carried by a lane deviation increase in the wide road condition at the highest n-back level. The problem-state-bottleneck model (triangles in Figure 7) predicts an effect of lane width on lane deviation as the model deviates more from the lane center in the highway condition. While predicting an increase of lane deviation across n-back levels in the construction condition, which is not present in the human data, the problem-state-bottleneck model predicts the effect of lane width closely with regard to human behavior (*RMSE* = 0.15, *R*^2^ = 0.14). In contrast, the central-bottleneck model (circles in Figure 7) shows no effect of n-back level, but overestimates the effect of lane width on lane deviation while still underestimating the total lane deviation (*RMSE* = 0.22, *R*^2^ = 0.92). Although, the model results do not favor the problem-state-bottleneck model as clearly, we argue that it shows a slightly better fit as the effect of lane width reflects human behavior while the central-bottleneck model does not capture the effects of either condition well.

### Eye-Tracking Results

**Figure 8.**
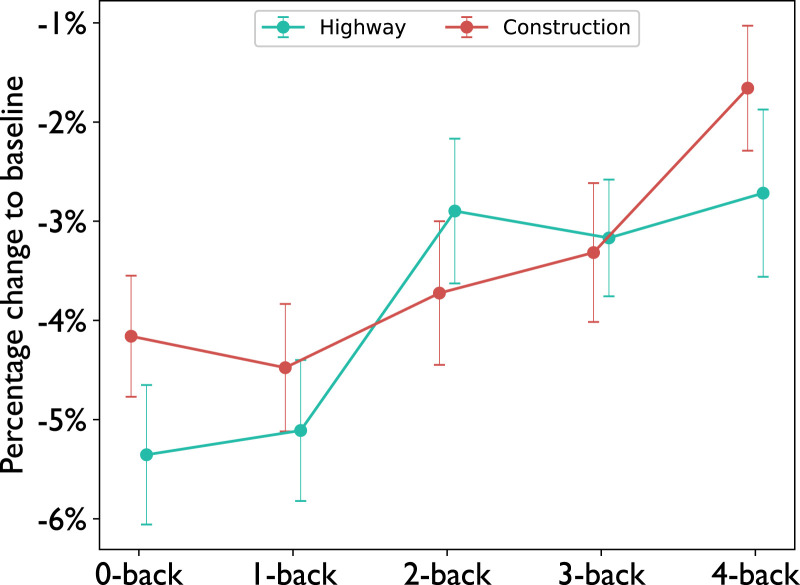
Percentage change to baseline of the pupil size across n-back levels. Error bars indicate the standard errors of the mean. The baseline pupil size was larger than the average pupil size leading to negative values on the y-axis.

**Figure 9.**
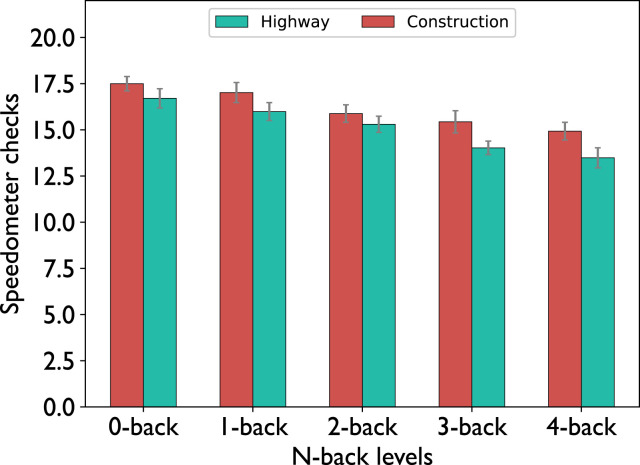
Number of fixations on the speedometer in between two consecutive speed signs. Error bars indicate the standard errors of the mean.

Before analyzing the performance in the n-back task, we assessed the task demand induced by the different levels of the task to validate the induced working memory load. Previous research has found increased pupil size as the contents in working memory increase suggesting that pupil size can be used as an indicator for working memory load (Kahneman & Beatty, 1966). Accordingly, analysis of the eye-tracking data revealed a significant increase in pupil size with respect to the baseline with increasing n-back difficulty $\left(F\left(4,21\right)=4.5,p=0.002,\eta_{p}^{2}=0.18\right)$ calculated by a 2-way repeated measures ANOVA. There was no interaction effect $\left(F\left(4,21\right)=0.69,p=0.6,\eta_{p}^{2}=0.03\right)$ and no difference could be found between driving conditions $\left(F\left(1,21\right)=0.83,p=0.37,\eta_{p}^{2}=0.04\right)$ (Figure 8). Furthermore, we analyzed the observed number of fixations on the speedometer in between two speed signs to assess how often participants monitored their speed to keep to the task-compliant speed. The data show a decrease with increasing n-back level $\left(F\left(4,21\right)=12.7,p<0.001,\eta_{p}^{2}=0.38\right)$ (Figure 9). While the participants seemed to show a numerically higher number of fixations on the speedometer in the construction condition compared to the highway condition, the difference was not significant according to a 2-way repeated measures ANOVA $\left(F\left(1,21\right)=1.3,p=0.26,\eta_{p}^{2}=0.06\right)$ and there was no interaction effect $\left(F\left(4,\;84\right)=0.71,p=0.58,{\;\eta }_{p}^{2}=0.03\right)$ .

### N-Back Performance

**Figure 10.**
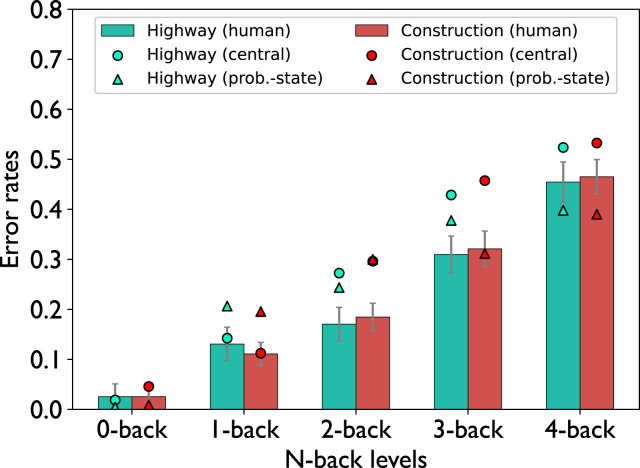
Errors in the speed regulation task (n-back task). Error bars indicate the standard errors of the mean.

As illustrated in Figure 10, participants made significantly more errors in the speed regulation task as n-back difficulty increased, which was revealed by a two-factor repeated measures ANOVA $\left(F\left(4,84\right)=30.35,\;p<0.001,\;\eta_{p}^{2}=0.59\right)$ . However, no significant differences in speed errors between lane widths $\left(F\left(1,21\right)=0.03,\;p=0.85,\;\eta_{p}^{2}<0.01\right)$ or interaction effects between n-back and lane width were observed $\left(F\left(4,84\right)=0.18,p=0.95,\eta_{p}^{2}<0.01\right)$ .

In addition to the matching effect in the driving performance, both the central-bottleneck model (*RMSE* = 0.08, *R*^2^ = 0.95) as well as the problem-state-bottleneck model (*RMSE* = 0.07, *R*^2^ = 0.83) mirror the effect of n-back difficulty on the performance in the speed regulation task with respect to human participants: as n-back difficulty increases, the model performed worse in the n-back task. However, the models overestimated this effect for higher n-back difficulties and made more errors than human participants. There is no effect of visuospatial demands on n-back performance in the models. As both models exhibit similar behavior regarding n-back performance, they represent human behavior equally well.

## Discussion

In this study, we investigated whether a bottleneck at a task-specific resource or a bottleneck at a task-unspecific resource could better account for interactions between working memory and visuospatial attention during driving. Scheunemann et al. (2019) proposed that the interactions could be due to a common resource at a task-specific level that needed to be accessed by both tasks (e.g., working memory) or due to a common resource, which is independent of the tasks (e.g., the central executive in Wickens’ model (Wickens, 2002)). We contrasted the two hypotheses by developing two cognitive models implementing these hypotheses. The central-bottleneck model is restricted by a bottleneck at a task-unspecific resource. The problem-state-bottleneck model implements a bottleneck at a task-specific resource (i.e., the problem state). We evaluated the behavior of the models by comparing their behavior to data collected in a dual tasking driving experiment. Our study showed that the effect of working memory on driving performance cannot be adequately categorized by a contention at a task-unspecific resource like the central processing unit, due to the relatively poor fit of the central-bottleneck model to human data with regard to driving performance. Instead, the models suggest an interaction at a common, task-dependent resource like the problem state, representing working memory usage, which is indicated by the problem-state-bottleneck model accounting for human driving behavior across both working memory load and lane width.

Regarding steering behavior, both models showed increased SRRs in the construction condition, which is in line with the behavior of human participants in the current study as well as with previous research that showed that SRRs increase with the task demand as long as the task is within capacity of the mental resources (Greenshields, 1963; Macdonald & Hoffmann, 1980; Mclean & Hoffmann, 1973; Scheunemann et al., 2019). However, these findings are highly dependent on the exact nature of the task, as reduced SRRs have also been shown when drivers performed a secondary task, while driving difficulty remained constant (Macdonald & Hoffmann, 1977). Macdonald and Hoffmann (1980) suggested that steering reversals increase with additional effort being put towards the driving task, which increases as the task demand in the driving task rises. However, when a secondary task exceeds the maximum mental capacity, mental resources (e.g., attention) are divided and are then drawn away from the driving task. Consequently, the effort being put into the driving task decreases, which results in a lower number of steering reversals. This effect is nicely demonstrated by the two models. In both models, steering control is modeled by the implementation of two control loops: high control vs. low control. The low-control loop does not update the steering position via the near and far point but merely checks whether the car is in an unsafe position in the lane and thereby models driving with low effort. In the construction condition the model remains in the low-control state for less time as high control is initiated when the model approaches the lane edges, which are closer to the car if the lane is narrower. This mechanism reflects the hypothesized behavior of human participants that driving in a construction site requires more effort and, thus, more resources.

However, while mirroring human behavior across lane widths, the central-bottleneck model falls short in predicting human behavior as it cannot account for the decrease in SRRs over increasing n-back levels. In contrast, the problem-state-bottleneck model implies that the problem state is needed even for a well-practiced control task such as driving and, therefore, predicts a decrease in steering operations over n-back levels. From human pupil data, we can conclude that the task demand increases with n-back level because we observed an increase in pupil size indicating higher workload. This is also reflected by human participants committing more errors across n-back levels as more speed signs have to be stored in working memory for longer periods of time. Furthermore, we observed that participants showed a decreasing number of fixations on the speedometer as the n-back difficulty increased. This further indicates that participants have less time available to invest in monitoring the car’s speed due to the increasing task demand of the n-back task. Thus, it can be argued that with increasing difficulty in the n-back task, the task draws away more resources from the driving task resulting in less time spent on the driving task in human participants.

The two models can account for this behavior in two ways via the different bottlenecks and their respective costs. In the central-bottleneck model, the competition for initiating production rules increases with increasing n-back level as higher n-back levels initiate more productions to recall speed signs further back in time. However, the cost of the central bottleneck is relatively low overall, leading to a low effect on steering reversal rates. In the problem-state-bottleneck model, driving is dependent on the problem state meaning that more resources between the two tasks are shared. Consequently, fewer processes can be executed in parallel and the n-back task draws away more time from the driving task when the n-back level increases resulting in fewer steering reversals across n-back levels in the problem-state-bottleneck model. This higher cost of the bottleneck is evident when comparing the total delay of steering updates caused by the problem-state bottleneck as n-back levels increase. This explanation is in line with threaded cognition, which claims that both tasks share the mental resources and an increased effort in the working memory task draws away resources from the driving task resulting in less time spent overall on steering control. According to this theory, the addition of a secondary task will result in fewer steering reversals as the central processing unit acts as a bottleneck for any two tasks that are performed simultaneously.

Lane deviation in human participants showed a similar pattern as observed in previous studies, with lane deviation decreasing in narrower lanes (De Waard et al., 1995; Dijksterhuis et al., 2011; Godley et al., 2004). However, no clear pattern emerged across working memory load levels. Previously, lane deviation has shown to *decrease* with increasing working memory load (Brookhuis et al., 1991; He et al., 2014), although Unni et al. (2017) reported no changes across n-back levels using the same experimental design as we did. Both models in our study capture the effect of increased lane deviation in the highway condition. In addition, the problem-state-bottleneck model also predicted an effect of increased lane deviation across n-back levels in the construction condition, which is not present in the human data. The effect between road conditions in the models is due to the increased time in the low-control loop in the highway condition, which does not adjust the steering to move back to the center until a critical distance to the lane edge has been reached. With regards to the effect of working memory load, the increased time spent on the n-back task leads to less time in the high-control loop, which further increases lane deviation, similarly to steering reversals. Similarly, as the driving productions cannot be initiated while the problem state is occupied, which leads to fewer steering productions, lane deviation is higher in the problem-state-bottleneck model when compared to the central-bottleneck model. This effect primarily affects the construction condition as the contention for resources is stronger in this condition. As the model spends more time in the high-control loop, the effect of the working memory task, which restricts time in the driving loop, becomes stronger.

Interestingly, neither the models nor the steering behavior of the human participants showed an *interaction* between the two manipulated concepts suggesting that the found interactions by Scheunemann et al. (2019) are exclusively present at the brain level, but not at the behavioral level.

Overall, the problem-state-bottleneck model captured human steering behavior across different task conditions and could account for different kinds of induced workload. In the future, well-tuned cognitive models that predict human behavior in different driving situations could support human-machine integration as proxies for humans in automated vehicles. Automated vehicle approaches, like “adaptive automation” (Hancock et al., 2013), could especially benefit in this regard as they attempt to adjust the level of automation in the vehicle to the operator’s mental load. The authors envision a system that monitors the driver’s cognitive state measured by brain activity or physiological sensors to determine periods of high cognitive workload. In these situations, the envisioned system could intervene to alleviate the driver’s cognitive workload. The objective in this endeavor is for the driver to remain attentive and actively engaged in the driving task but re-adjust the driving responsibilities when the driver’s safe handling of the situation cannot be guaranteed or when the automated system is failing. In an ideal system, the driving task should be optimized such that the driving responsibilities are matched to the general and momentary capabilities of the individual driver. Although such a system does not yet exist, the idea has been evaluated before using brain measurements to classify frustration in human drivers, which showed significant safety gains (Damm et al., 2019). While this research is promising, it is currently unclear how much mental workload is induced by the task demands that are part of everyday driving leading to high uncertainties in the classification of the human state. Consequently, which driving responsibilities should be automated, which should be performed by the human and what the mode of intervention needs to look like remains largely unspecified to this date.

A crucial step toward effective adaptive automation is to be able to accurately assess cognitive workload while driving, in order to know when the system should adapt the driving responsibilities assigned to the human operator. While predicting cognitive workload can potentially be done using physiological measures like heartrate variability or pupillary response (Kahneman & Beatty, 1966) and even neuroscientific methods like electroencephalography (EEG; e.g., Aricò et al., 2016; Scerbo et al., 2003) or functional near infrared spectroscopy (fNRIS; e.g., Bunce et al., 2011; Unni et al., 2017), these methods are limited because overall workload is driven by multiple external factors which interact at the brain level. Interactions between different factors have the potential to considerably deteriorate the accuracy of the system as was evidenced by Scheunemann et al. (2019) constituting a real challenge in brain-based adaptive automation technologies.

Herein lies the great potential of using explainable computational models as showcased in this work. The model we present here provides not only behavioral predictions during multi-factorial driving but it can also be used to disambiguate how different factors contribute to a degradation of driving performance, for example, by a bottleneck in working memory. As such, it can be used to infer modes of intervention, which alleviate the mental load of the driver by eliminating crucial points of failure in cognitive processing. Furthermore, since cognitive models are inherently explainable in their predictions, they potentially transfer well to hitherto unseen situations, which can be helpful to constrain the output of conventional black-box models used in previous work (Damm et al., 2019). For example, in a stressful highway situation, which might potentially overload the driver, the ACT-R model could be used to identify concrete assistance to the driver such as increased steering automation or highlighting information that might otherwise be stored in working memory.

### Limitations

An important point to mention is the overestimation of the steering reversal rates by the problem-state-bottleneck model in the 0-back task (Figure 6), which represents normal driving conditions. To understand why this happens, we repeat that in the 0-back, similarly to higher n-back levels, the chunk that encodes the target speed is transferred from the visual buffer to the problem state buffer after a speed sign is encountered. However, we categorized the target speed as control information in the problem-state-bottleneck model meaning that it was eventually stored in the goal buffer. This means that once the information is recalled and stored in the goal buffer, there is no need to retain the information in the problem state and it can be instantly released causing the problem state to be free. With regards to the multitasking behavior of the problem-state-bottleneck model, this means that the bottleneck at the problem state is limited to the recall phase but not the rehearsal phase in the 0-back task, eliminating a large part of the interaction between the two tasks. This shortcoming of the model should be addressed in future models possibly by extending the model to involve a tighter control of the currently followed speed, which would impact the problem state in the ACT-R model. As performing the 0-back task likely requires working memory usage in human participants, evidenced by the non-zero error rate for this task condition, it is reasonable to include such a control mechanism in a future model. With steering control being dependent on the availability of the problem state, we estimate that this may show decreased driving performance in the model. However, as this was not the primary focus of this work, implementing and validating such a mechanism was out of scope.

## Conclusion

In this paper, we provided explainable predictions with regards to the interactions between visuospatial attention and working memory load during driving. These predictions could be used by future automation systems to disambiguate the effect of different external mental loads to evaluate when drivers are in need of assistive technologies. Further studies could attempt to predict brain activation to quantify the contribution of each subtask to overall workload on the brain level. Ultimately, this could lead to reducing mental load as a risk factor for driving and thereby to significant safety improvements in everyday traffic.

## Key Points

- Interactions between visuospatial attention and working memory load can be accurately modeled by a bottleneck at the problem state resource in ACT-R.
- A central bottleneck, which is independent of the specific tasks, does not reflect the impact of working memory load on driving performance.
- Drivers can perform fewer control actions with increased working memory load resulting in decreased steering performance.
- Interactions between visuospatial attention and working memory load do not show at the behavioral level.

## Appendix

**TABLE A1:** Model Parameters

Parameter	Central	Problem State
*Rt*	−100	−100
*Mp*	19	24
*Ans*	0.4	0.5
*Lf*	1.0	0.3
*Dist*	0.7	0.75
*Thc*	3	3
*Scale*	0.6	0.925
*k*_*thw*_	16	16 * scale
*k*_Δ*thw*_	4	4 * scale
*k*_*far*_	0	0
*k*_*near*_	3	3
Θ_*nmax*_	0.07	0.03

The middle column displays the values that have been selected for the central-bottleneck model, whereas the right column displays values that have been fit for the problem-state-bottleneck model. The top four values are ACT-R memory parameters and determine the accuracy (*rt*, *mp*, *ans*) and the speed (*lf*) of items being recalled. The bottom parameters are general driving (*dist*, *thc*) and steering parameters (*scale*, *k*_*thw*_, *k*_Δ*thw*_, *k*_*far*_, *k*_*near*_, Θ_*nmax*_) that constrain the lateral and longitudinal control functions.
